# Recent advances in understanding body weight homeostasis in humans

**DOI:** 10.12688/f1000research.14151.1

**Published:** 2018-07-09

**Authors:** Manfred J. Müller, Corinna Geisler, Steven B. Heymsfield, Anja Bosy-Westphal

**Affiliations:** 1 Institute of Human Nutrition and Food Science, Christian-Albrechts-Universität zu Kiel, Kiel, Germany; 2 Pennington Biomedical Research Center, Baton Rouge, LA, USA

**Keywords:** body weight homeostasis, fat mass, obesity, energy balance

## Abstract

Presently, control of body weight is assumed to exist, but there is no consensus framework of body weight homeostasis. Three different models have been proposed, with a “set point” suggesting (i) a more or less tight and (ii) symmetric or asymmetric biological control of body weight resulting from feedback loops from peripheral organs and tissues (e.g. leptin secreted from adipose tissue) to a central control system within the hypothalamus. Alternatively, a “settling point” rather than a set point reflects metabolic adaptations to energy imbalance without any need for feedback control. Finally, the “dual intervention point” model combines both paradigms with two set points and a settling point between them. In humans, observational studies on large populations do not provide consistent evidence for a biological control of body weight, which, if it exists, may be overridden by the influences of the obesogenic environment and culture on personal behavior and experiences. To re-address the issue of body weight homeostasis, there is a need for targeted protocols based on sound concepts, e.g. lean rather than overweight subjects should be investigated before, during, and after weight loss and weight regain. In addition, improved methods and a multi-level–multi-systemic approach are needed to address the associations (i) between masses of individual body components and (ii) between masses and metabolic functions in the contexts of neurohumoral control and systemic effects. In the future, simplifications and the use of crude and non-biological phenotypes (i.e. body mass index and waist circumference) should be avoided. Since changes in body weight follow the mismatch between tightly controlled energy expenditure at loosely controlled energy intake, control (or even a set point) is more likely to be about energy expenditure rather than about body weight itself.

## Introduction

For decades of research, body weight control has been taken as given and scientists have looked at different aspects of a proposed biological control system including genes, neuropeptides, hormones, proteins, and metabolites. However, without being negative at all, our present knowledge and concepts of body weight control explain neither weight gain in individual subjects nor the obesity epidemic in populations. Thus, our aim here is to show that we might do better with a new way of thinking and a different approach.

## Conceptual framework

The conceptual framework of a biological control of body weight is mainly based on animal studies (for a very recent and excellent review, see 1). For example, when compared with feeding a standard chow (or mixed) diet, feeding rats with an energy-dense diet (rich in fat and sugar) resulted in overeating and a disproportionate weight gain ^2^. After withdrawing that diet and introducing a mixed diet again, rats then spontaneously returned to the weight of continuously mixed-diet-fed control rats ^2^. *Vice versa*, after caloric restriction and weight loss, rats regained body weight with re-feeding, reaching their previous track of weight gain again ^3^. These findings are taken as evidence for an inherited body weight (or in rats as an inherited weight gain with age) and served as examples of a so-called “set point”.

The set point theory assumes a strong genetic and humoral control of body weight characterized by a proportional feedback system designed to control body weight (or body energy and/or fat and/or protein and/or glycogen) to a constant “body-inherent” weight (or “body-inherent” energy, fat, protein, or glycogen content, respectively). The control systems (or thermo-stats, lipo-stats, proteo-stats, or gluco-stats, respectively) adjust food intake and/or energy expenditure (EE) in proportion to the difference between the current and the set point weight (for a previous F1000 Faculty Review, see 4). Although its molecular nature is still unknown, the set point paradigm is popular among molecular biologists, and today it is textbook knowledge. It is assumed that set points are defended by biological mechanisms within the brain stem and the hypothalamus. This is part of the homeostatic system controlling energy intake (EI), EE, energy stores (ES), and thus energy balance (EB) involving (i) afferent signals from the periphery, like leptin-signaling ES in adipose tissue to control EI, and (ii) efferent signals, like the sympathetic nervous system (SNS) activity to control EE. Accordingly, a defect in the lipo-static control system characterized by leptin resistance is considered to result in hyperphagia and relative or absolute hypometabolism and thus to explain obesity ^1^.

Homeostatic control of body weight keeping to a set point is thought to be under genetic influences. The genetic component also integrates multiple ancestral influences like growth and pubertal development ^5^. Developmental influences add to the differences in body weight (and body composition) between individuals by affecting the tightness of the homeostatic processes involved in body weight control ^6^. Growth patterns in early life also add to susceptibility of certain diseases and mortality and thus the cardio-metabolic risk. Trans-generational effects on body weight are also reflected by the observation that in primates the trend in birth weight lags generations behind the trend in maternal weight ^7^.

During the last few decades, research activities mainly focused on the biology of the feedback loop between adipose tissue and the hypothalamic melanocortin neuronal system mediated by leptin controlling EI and EE ^8, 9^. The melanocortin system in the basomedial hypothalamus is highly sensitive to nutrient availability, including the leptin signal. Leptin is secreted from adipocytes in proportion to fat mass (FM) and adiposity. In addition, with increasing adiposity, hyperinsulinemia and insulin resistance develop. Both leptin and insulin sensitivity moderate the strength of the association between FM and the body weight control system. Central and peripheral resistances to leptin and/or insulin (as seen in obese patients) are considered to reduce their effects on EI ^8, 9^.

Leptin as well as insulin bind to specific receptors in the brain. These receptors are found not only in the hypothalamus and brain stem but also in pre-frontal regions, the hippocampus, and the amygdala, all together explaining the multifaceted effects of leptin and insulin not only on EB but also on learning, memory, and rewards ^9^. Obviously, this feedback system goes beyond a homeostatic control of body weight (based on the body’s needs and and/or its deficits of energy and specific nutrients) and refers to non-homeostatic factors (i.e. environment, hedonics, palatability, opportunity, cognition, learning, and social factors) too. Thus, EI (and also appetite, hunger, and satiety) is explained by both homeostatic and non-homeostatic factors.

As a modification of the classical set point concept, an asymmetric (or threshold) body weight control system has been proposed ^8, 10^. The idea is that the anabolic response to leptin becomes evident only when plasma leptin levels have fallen under a certain threshold level, which may resemble a low set point related to starvation and the risk of death ^8, 10^. In this model, no biological control is assumed to exist with overfeeding.

When compared with the set point paradigm, an alternative model to explain changes in body weight involves multiple “body weight steady states”. With overfeeding and underfeeding, weight changes result from the difference between EI and EE. During weight gain or weight loss, the differences between EI and EE diminish more and more owing to increases or decreases in fat free mass (FFM) and EE and metabolic adaptations (in response to weight loss), and a new stable lower or higher body weight is finally reached, reflecting a zero EB (i.e. there is no difference between EI and EE anymore). This new steady state in body weight is called the “settling point” ^1, 4^. In this model, there is no need for feedback control of either EI or EE. It is worthwhile to mention that the settling point model does not take into account metabolic adaptations to changes in EI and body weight (see below; 1).

The settling point paradigm also relates to body composition. This is because weight changes follow changes in body composition and the energy density characteristics of individual body components ^11– 14^. Any energy imbalance is partitioned between stored or mobilized fat in FM and protein and glycogen in FFM ^12^. In a healthy subject, about 70–85% and 15–30% of body weight changes are due to FM and FFM, respectively. These numbers differ throughout the course of weight loss with a greater loss in FFM in the early phase while the loss of FM exceeds decreases in FFM during ongoing weight loss ^13^. Partitioning of FM and FFM with weight changes is affected by age and exercise. However, even during controlled overfeeding and underfeeding of young healthy subjects, there is a considerable inter-individual variance in the fraction of energy imbalance from or to FM and FFM, respectively ^13, 14^.

The variance in partitioning adds to the inter-individual variance in weight change at a given EB. This is due to the differences in the energy content (or energy densities) of FM (9.4 kcal/g) and FFM (1.8 kcal/g) ^12^. For example, at an energy imbalance of 940 kcal/day, it will take about 10 days to lose or gain 1 kg of FM at a 100% fraction to or from FM, whereas theoretically one may gain or lose 500 g of FFM per day at a 100% fraction of energy imbalance to or from FFM.

With ongoing weight loss, the proportion of FM exceeds that of FFM, resulting in an increased proportion of FFM relative to FM after weight loss ^11, 13, 14^. This impacts EI and EE and thus EB and body weight ^15– 18^. The drive to eat is related to the energy demand of FFM, but putative energy-demanding signals from skeletal muscle and from high-metabolic-rate organs like the liver, kidneys, heart, and brain still remain to be characterized. FFM is closely related to resting EE (REE; ^17^), and both REE and FFM are determinants of EI, hunger, and self-selected meal size ^15, 16, 18^. With weight loss, REE and FFM decrease at a concomitant change in FFM composition with a disproportional loss in skeletal muscle mass compared with visceral organs like the liver and kidneys ^13, 14^. Although the neuroendocrine link between FFM and/or FFM composition and EI has not been characterized up to now, it is tempting to speculate that this may affect appetite and hunger feelings. With weight regain, FFM is increased together with a disproportional increase in FM (i.e. FM and FFM cannot change independently from each other ^19^). The increase of FFM then aims to increase REE and thus to finally match EI and EE. In fact, hyperphagia related to FFM depletion persists until full recovery of FFM, and thus a new steady state is reached ^15, 19^. However, this will also increase EI again and so may end in some kind of a roundabout with no escape, which argues against the theory. Although signals generated in FFM affecting EI have to be identified in the future, the general idea of FFM as a determinant of EI brings FFM into the center of “body weight control” with the faster recovery of FM as a so-called “collateral fattening” phenomenon ^19^. Then putting FFM within the center of the discussion ^15, 16, 18, 19^ is an alternative or additive concept when compared with most of the recent research activities on body weight control with a primary focus on FM ^8, 9^.

Both the set point and the settling point paradigms do not address possible “gene-by-environment” interactions and metabolic adaptations. Therefore, the set point paradigm has been further elaborated by an alternative concept proposing a so-called “dual intervention model” ^1, 20^. In this model, there is no single set point and body weight may change in response to environmental factors within upper and lower “intervention points” (or upper and lower boundaries) where the boundaries themselves and/or the distance between them may be biologically (e.g. genetically) determined ^1, 20^. In this model, the lower boundary is considered to reflect the risks of starvation, wasting diseases, and survival, whereas the upper boundary is related to the risk of predation ^1, 20^. The model relates to the original concept of Castro and Plunkett ^21^, where EI is controlled by uncompensated (primarily environmental) as well as compensated (i.e. biological) factors. While the former factors are unaffected by EI, only the latter have negative feedback loops ^1, 20, 21^. Interestingly, uncompensated or environmental factors may override biological control, which thus seems to be loose in response to overfeeding but is tight in response to weight loss. The dual intervention model combines the set point (feedback control of body weight at the two boundaries only) with the settling point paradigm (explaining flexible weight changes between the boundaries ^1, 20^).

It is likely (but not proven) that the two intervention points are regulated separately ^1^. However, other authors ^22^ have recently proposed that the upper and lower boundaries are linked together, switching between the resting state and feeding. It is then tempting to speculate that at the population level fatness may change symmetrically. This idea does not fit with the asymmetrical distribution of body mass index (BMI) seen in Western populations ^1^. Anyhow, if one assumes an asymmetric control of body weight ^8, 10^, the meaning of the two boundaries may differ, with the lower boundary explaining resistance to weight loss in both normal-weight and obese subjects and the upper boundary being variable or even weak to defend body weight against overfeeding. It has been speculated that the level at which control mechanisms become activated is genetically controlled and thus may show a considerable inter-individual variance ^1, 20^.

To summarize, at present there is no consensus framework to explain body weight control in humans. Presently, there are three different models which have been developed from animal data. First, a set point suggests (i) a more or less tight and (ii) symmetric or asymmetric biological control of body weight. Alternatively, a settling point rather than a set point has been proposed with adaptations of body weight to energy imbalance without any need for feedback control. Finally, the “dual intervention point” model combines both paradigms. All models presently serve as a possible conceptual framework in research on physiology and cellular biology of body weight control. However, body weight homeostasis may be overridden by the influences of the obesogenic environment and culture, which have a considerable impact on personal behavior and experiences and thus are considered as the major drivers of the obesity epidemic ^23^. However, these concepts suggest that in humans living in affluent societies, biological control of body weight is unlikely to become apparent.

## What is the human evidence of body weight control?

In humans, the proof of the matter mainly refers to observational data obtained in large populations as well as to interventions in normal-weight subjects and in obese patients. By contrast, rigorously well-controlled experiments addressing body weight homeostasis in humans rarely exist. As far as observational data in free-living subjects are concerned, it was and still is impossible to control for all variables, weakening any conclusion. In addition, most observations are based on cross-sectional study designs which do not allow far-reaching conclusions. Then, the interpretation of the data is down to the intelligence of the scientists and to what they want to be true. This is a critical point of the present discussion.

### Monogenetic forms of obesity, heritability estimates, and genome-wide association studies supporting the idea of a biological control of body weight

During the last 30 years, specific study designs (e.g. twin, family, and adoption studies) have been used to calculate the total genetic influence on body weight (for a recent review, see 24). More recently, genome-wide association studies (GWAS) on BMI, waist circumference (WC), and FM have been undertaken with the goal to identify human genes that biologically cause overweight. While familial correlation in BMI is high in monozygotic twins, so far GWAS on obesity at the population level explained a minor proportion of the variation in adult BMI only.

Up to now, a total number of 19 rare monogenetic defects associated with severe obesity are impressive manifestations of disturbed control of body weight ^25^. These rare cases are in favor of the idea that specific genes influence EI and/or EE and thus EB and body weight. However, these findings do not explain population-wide obesity. Although specific variants in individual genes (e.g. the FM- and obesity-associated gene, *FTO*) are considered suitable candidates to explain the individual variability in (i) the predisposition to become obese or (ii) individual responses to weight loss strategies in obese patients, the proposed genetic basis of obesity is still uncertain ^24^.

Using specific study designs (e.g. twin, family, and adoption studies), heritability ^1^ estimates (which are considered synonymous with genes) of BMI, FM, and visceral adipose tissue (VAT) have been calculated in numerous studies, which gave evidence for a biological influence on body weight ^26– 41^. The familial correlations in BMI were between 0.20 and 0.23 in parent–offspring pairs and 0.20 and 0.34 in dizygotic twins and reached 0.58 to 0.88 in monozygotic twins. In general, additive genetic factors explaining the proportion of variation in BMI varied between 0.31 and 0.85. Molecular mechanisms of heritability may not be limited to DNA sequence differences, since epigenetic factors also contribute to the phenotype. In fact, analyzing DNA-methylation profiles in pairs of monozygotic and dizygotic twins may be due to epigenomic differences in the zygotes adding to heritability estimates ^42^.

When different age groups of twin pairs were compared, the proportion of BMI explained by genetic and epigenetic factors increased until late adolescence with no or only minor effects of the shared environment ^40^. By contrast, shared environmental factors related to education and/or culture seemed to have a stronger influence during mid-puberty. Furthermore, in pooled cohorts of a total of 140,379 complete twin pairs from different regions of the world, the heritability estimates of BMI decreased from 0.77 in young adults to 0.59 in adults aged 70 to 80 years, which was independent of the obesity prevalence in the populations studied ^41^. However, heritability estimates cannot explain steep increases in the prevalence of obesity, which are due to non-biological and thus environmental changes as the driving factors. Furthermore, heritability does not take into account the complexity of the genotype–phenotype relationship. In addition, additive and non-additive genetic effects cannot be addressed separately. Finally, incomplete adjustments for co-variates like growth spurts during puberty or regional diversities in the environments affect heritability estimates.

In addition to cross-sectional data from population studies, differences in the response to overfeeding studied in pairs of monozygotic twins showed that inter-pair variances in gains of weight, FM, and VAT were found to be three to six times higher than the respective intra-pair variance ^26, 27^. This was taken as evidence for a “genotype-overfeeding interaction” that determines weight and fat gain as well as fat distribution. *Vice versa* with negative EB (due to a controlled exercise program ^31^), the intra-pair variances in changes in weight, FM, and VAT were also lower than the inter-pair variances, suggesting a “genotype-underfeeding interaction” as well. However, these data have to be compared with considerable intra-individual variances in changes of body weight, which have not been taken into account in the studies cited ^24^.

The high heritability estimates were not supported by the results of recent GWAS on BMI, FM, or VAT in greater populations ^24^. Up to now, 115 genetic loci have been identified where sequence variation was statistically associated with the BMI, explaining 2 to 3% of the variation in adult BMI only ^43^. In addition, longitudinally, no significant associations were found between any lead single nucleotide polymorphisms (SNPs) and weight changes ^44^. Obesity thus has a polygenic architecture, with small effects of each associated gene ^5^. The polygenic basis of adiposity may provide small sensitivities to environmental influences. More recent data suggested that obesogenic environments accentuate the genetic risk of obesity ^45^: using BMI as an outcome and a 10-variant genetic risk score in a socially deprived population, researchers found that genetic risk was associated with 3.8 kg extra weight in a normal subject. These data were compared with 2.9 kg extra weight in the least-deprived group ^45^. This finding may be taken as evidence for a moderate gene–obesogenic environment interaction.

### Methodological limits do not allow detailed insights into body weight control

It has been stated already that it is impossible to directly assess a set point in humans ^46^. Even in controlled experiments (e.g. using overfeeding or underfeeding protocols), EI and EE cannot be tightly controlled, owing to compensations on both sides of the EB and the components of EB being dynamically inter-related. This issue became obvious in a controlled underfeeding and overfeeding protocol in healthy normal-weight subjects ^13^. Comparing the differences between (i) the EB data calculated from the difference between EI and EE and (ii) EB data based on changes in accurate measurements of FM and FFM (assuming their constants for energy equivalents) as an alternative approach resulted in considerable discrepancies between the two measures of EB of about 800 to 1,000 kcal/day ^47^. These discrepancies cannot be explained by the limited precision of the methods used to assess either EI or EE or body composition only ^47^.

Although EI is considered as the major driver of individual weight gain and the population-wide obesity issue ^23^, it cannot be measured with confidence ^48^. There are large errors involved in the methods used under everyday living conditions to assess EI at the individual as well as the population level ^48, 49^; thus, true variations and between-group differences cannot be differentiated from measurement errors. Today, the measurement of 24-hour EE (by DLW) together with direct assessments of FM and FFM are considered the most precise and valid way to investigate EI during periods of more than 3 to 4 weeks ^48, 50, 51^. However, this approach does not provide detailed data on food and nutrient intakes. Hence, the great dilemma of nutrition research is obvious.

It is obvious that limitations in both our present concepts of biological control and methods used to assess the individual components of EB limit the direct assessment of a biological control of body weight in humans.

### Observational data questioning a biological control of body weight in humans

Globally, about 20 to 25% of adult populations are presently obese ^52, 53^. When compared to the previous generation, there was a more than twofold increase in the prevalence of obesity in affluent societies. This is indirect evidence for the idea that a tight control of body weight is unlikely to exist in humans living in an obesogenic environment. Alternatively, the obesity epidemic is presumably due to environmental, societal, and economic “drivers” rather than the proposed biological determinants of body weight ^20, 23^.

A tight biological control of body weight is also questioned by repeated measurements of body weight, which show a considerable intra-individual variance in its spontaneous day-to-day changes ^13, 24^. Even during controlled underfeeding and overfeeding, there are high intra-individual day-to-day variances in weight loss and weight gain which resemble inter-individual variances in weight changes ^13, 24, 54^. However, when compared with weight loss, gaining body weight is slow, suggesting again that body weight is, to a certain degree (almost not perfectly), defended. It has been proposed that this defense may constrain weight changes ^55^.

As far as weight-reduced obese patients are concerned, only 20% maintain at least 10% weight loss over a period of 1 year, suggesting that in free-living trials weight loss maintenance is difficult to hold ^56^. This is also true in lifestyle intervention trials, such as the Diabetes Prevention Program ^57^ and the Look AHEAD Study ^58^. In the "Biggest Loser Competition", an extreme weight loss was observed with 58 kg at the end of the competition ^59^. However, the regain was 41 kg after 6 years of follow up ^59^. This regain was taken as evidence for the set point paradigm. In addition, long-term adaptation and a stable body weight after weight loss following bariatric surgery has been proposed to reflect a permanent re-setting of the body weight set point, restoring “normal” leptin signaling (or its downstream signals) in the hypothalamus ^60, 61^. Accordingly, after bariatric surgery, the proposed re-programming of the body weight defense mechanisms at a lower body weight was not associated with increased hunger feelings or reduced EE ^61^. By contrast, animal data suggest no increase in hypothalamic leptin sensitivity after weight loss due to bariatric surgery, questioning the idea of re-programming a set point ^60^.

Thus, observational studies on greater populations living in affluent societies and also clinical data on obese patients do not provide consistent evidence for a biological control of body weight.

## Aspects of body weight homeostasis to be addressed in future studies

The present issues related to biological control of body weight in humans are due to preliminary and simplifying concepts of biological control of body weight, weak study designs (i.e. cross-sectional observational studies indicating associations only), methodological issues associated with the assessment of EI, EE, and EB, and/or inappropriate phenotypes studied so far (i.e. the BMI). Alternatively, biological control of body weight may exist but may not become apparent in subjects living in an obesogenic environment supporting a lifestyle characterized by a high EI at low physical activity. Accordingly, several points should be addressed in future studies.

### Need of studying normal weight instead of overweight subjects

Following the framework of the “dual intervention model” ^1, 20^, biological control of body weight may be overridden by strong environmental and economic drivers (i.e. uncompensated factors) of overweight. Thus, studying overweight subjects in affluent societies is unlikely to address biological control (if it exists). Alternatively, body weight homeostasis should be investigated in lean subjects undergoing weight gain, weight loss, and weight maintenance.

### Crude phenotypes should not be addressed in research anymore

Investigating crude phenotypes like BMI and WC (i.e. phenotypes which have been most frequently used in studies on heritability estimates as well as in GWAS) is spurious and cannot provide any deep insights. This is because BMI and WC are merely surrogate measures of nutritional status. BMI is calculated from weight and height squared and has no biological meaning. Both BMI and WC have practical value in daily clinical practice. By contrast, they are weak outcomes in research on body weight homeostasis ^62– 64^. Furthermore, there are ethnic differences in the associations between these crude measures and FM, FFM, or VAT which lead us to question their value as a phenotype to be used in multicenter studies on subjects with different ethnic backgrounds ^65^. It is worthwhile to remember that the concept of BMI dates back to a period of underdeveloped scientific methodologies and simplistic theories ^63, 64^. By contrast, at the time of modern biomedical science, still keeping to BMI and/or WC is unacceptable.

Alternatively, addressing a suitable phenotype, a framework is needed to assess the structures of the body ^66, 67^. To do so, we should start with a simple question: what do we really want to know? In the case of obesity, we are interested in excess FM, which can be easily measured by, for example, bioelectrical impedance analysis (BIA) in population studies and/or by either densitometry (as assessed by air displacement plethysmography [ADP]) or dual energy absorptiometry (DXA) in clinical studies. Obesity-related cardio-metabolic risks are characterized by hyperinsulinemia (i.e. basal plasma insulin levels >7–10 μU/mL), hypertriglyceridemia, elevated biomarkers of inflammation, and high blood pressure. Alternatively, an estimate of liver fat (as measured qualitatively using ultrasound [US] or MRS, Magnetic Resonance Spectroscopy or by biochemical estimates, e.g. liver enzymes and fetuin A) or VAT (as measured by magnetic resonance imaging [MRI]) can be used as a risk estimate. As far as malnutrition is concerned, this is characterized by recent weight loss (in the case of wasting diseases) and/or a low muscle mass (i.e. sarcopenia, which is characterized by either DXA or MRI or multifrequency BIA validated against those two reference methods). Fluid overload is again measured with confidence using multifrequency BIA or D _2_O dilution. Finally, in the case of risk of osteoporosis, bone mineral density plus skeletal muscle mass are assessed by DXA measurements.

### A “multi-level–multi-systemic approach” should be used

Presently, it is unclear whether body weight control is about control of (i) the static masses of the body, including masses of individual organs and tissues (which add up to body weight), and/or (ii) the association between FM and FFM and their concerted changes when body weight changes. In any case, focusing on body mass or masses of organs and tissues alone does not take into account different levels and systems of control ^24^. Thus, to go on a more systemic approach to body weight homeostasis, we need to take into account different levels and systems of control ^67^.

**Figure 1.**
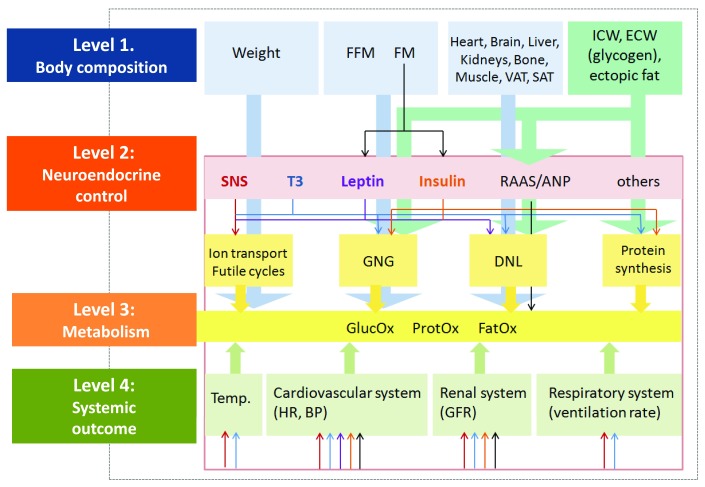
Proposed model of body weight control using a multi-level–multi-scale analysis based on its structural and functional determinants. Individual body components and their inter-relationships are seen in the context of metabolism, endocrine determinants, and systemic outcomes, e.g. body temperature, heart rate, etc. The model thus addresses relationships between organ and tissue masses (rather than their isolated masses only) in the context of age- and sex-specific metabolic or functional traits (e.g. energy expenditure, insulin sensitivity, muscle strength, and physical performance) together with the systemic response of the body. The model is supported by the findings that (i) changes in weight (during either weight loss or weight gain) are associated with concomitant changes in body composition, which are not independent of each other (e.g. FM and FFM both decrease with weight loss, while muscle mass decreases, whereas FM increases in the case of age-related sarcopenia) and (ii) body weight control hinges on the relationship between organs and tissues and their functional correlates. See text and ^67^ for further details. ANP, atrial natriuretic peptide; BP, blood pressure; DNL, *de novo* lipogenesis; ECW, extracellular water; FatOx, lipid oxidation; FFM, fat free mass; FM, fat mass; GFR, glomerular filtration rate; GlucOx; glucose oxidation; GNG, gluconeogenesis; HR, heart rate; ICW, intracellular water; ProtOX, protein oxidation; RAAS, renin-angiotensin-aldosterone system; SAT, subcutaneous adipose tissue; SNS, sympathetic nervous system; T3, 3,5,3'-triiodothyronine; Temp, body temperature; VAT, visceral adipose tissue.

Up to now, studies on body weight control in humans have addressed the structural level only (level 1 in Figure 1). According to a more advanced model ^67^, control is about relationships within and between structures, their related functions and systemic outcomes, and thus not about body weight or its individual components only. The masses of organs and tissues and their inter-relationships (level 1) have to be addressed in the contexts of neurohumoral control (level 2) together with metabolic (e.g. EE, i.e. level 3) and systemic outcomes (e.g. heart rate, blood pressure, respiration, excretion, and body temperature, i.e. level 4) ( Figure 1).

Following that “multi-level–multi-systemic” model, body weight control (if it exists) is likely to happen between, but not at, a single mass and level (i.e. control is about operating within and between levels). Then changes in body weight follow the control of the associations within levels (e.g. between FM and FFM or between individual organs and tissues) as well as between different levels (e.g. between structures and metabolic functions). Thus, strictly speaking, the issue of body weight control is about associations within and between different levels and systems which add up to maintain a stable body weight.

Consequently, phenotypes worthwhile of study are related to body mass–body function relationships rather than to body weight itself ^67^ ( Figure 1). The concept of functional body composition ^67, 68^ refers to, for example, the association between FFM (and its anatomical and physical characteristics) and REE in the context of neurohumoral control (e.g. thyroid state and SNS activity) and related systemic outcomes (e.g. heart rate and body temperature). Similarly, the association between FM (or its distribution) and plasma leptin levels has to be seen in the contexts of insulin resistance, O2 consumption and CO _2_ production, as a measure of lipid oxidation and respiration. To go on with that idea, the structure–function associations and their changes have to be studied separately in different situations, e.g. before, during, and after weight change as well as during weight maintenance. As far as weight loss is concerned, it has been shown recently that control systems involved in metabolic adaptation differ between weight loss and weight maintenance ^69^.

### Metabolic adaptations and compensations may provide a suitable phenotype to study

Adaptive thermogenesis (AT) refers to changes in EE which are independent from changes in FFM and FFM composition ^69– 73^. Since there is a considerable inter-individual variance, AT may provide a suitable phenotype to investigate in future studies. AT is asymmetric (i.e. during weight loss it adds to energy sparing, whereas no energy dissipation is observed with overfeeding ^70, 71^). AT is also observed after massive weight loss following bariatric surgery in severely obese patients ^72^. During early starvation, AT is related to hepatic glycogen depletion and the fall in insulin secretion ^13, 69^. Thus, AT is considered a metabolic adaptation to meet glucose oxidation in the brain (i.e. the brain's energy metabolism requires 80–100 g glucose per day ^69^). AT is associated with systemic outcomes, i.e. decreases in body temperature, heart rate, and glomerular filtration rate ^13^. These data suggest that AT is part of the concerted physiological response to weight loss, with the fall in insulin secretion as its major characteristic ^69^. In the long term, AT adds to weight loss maintenance ^69, 73– 75^ where AT is related to the low plasma leptin levels sparing triglycerides stored in adipose tissue where a low FM limits biological functions like reproduction ^69^. Obviously, the meaning of AT varies with the phase of caloric restriction and weight loss.

AT has been related to the set point and settling point paradigms. Recently, three models for AT have been proposed ^74^. First, a “mechanical model” related to the settling of body weight; second, a “threshold model”, where AT is related to the decrease in FM below a minimum (i.e. a set point); and, third, a so-called “spring-loading model” with an effect on the dynamics of weight loss. No model fully explained AT. However, during weight maintenance, decreases in REE were consistent with the threshold model and thus a low set point related to ES in adipose tissue and plasma leptin levels.

**Figure 2.**
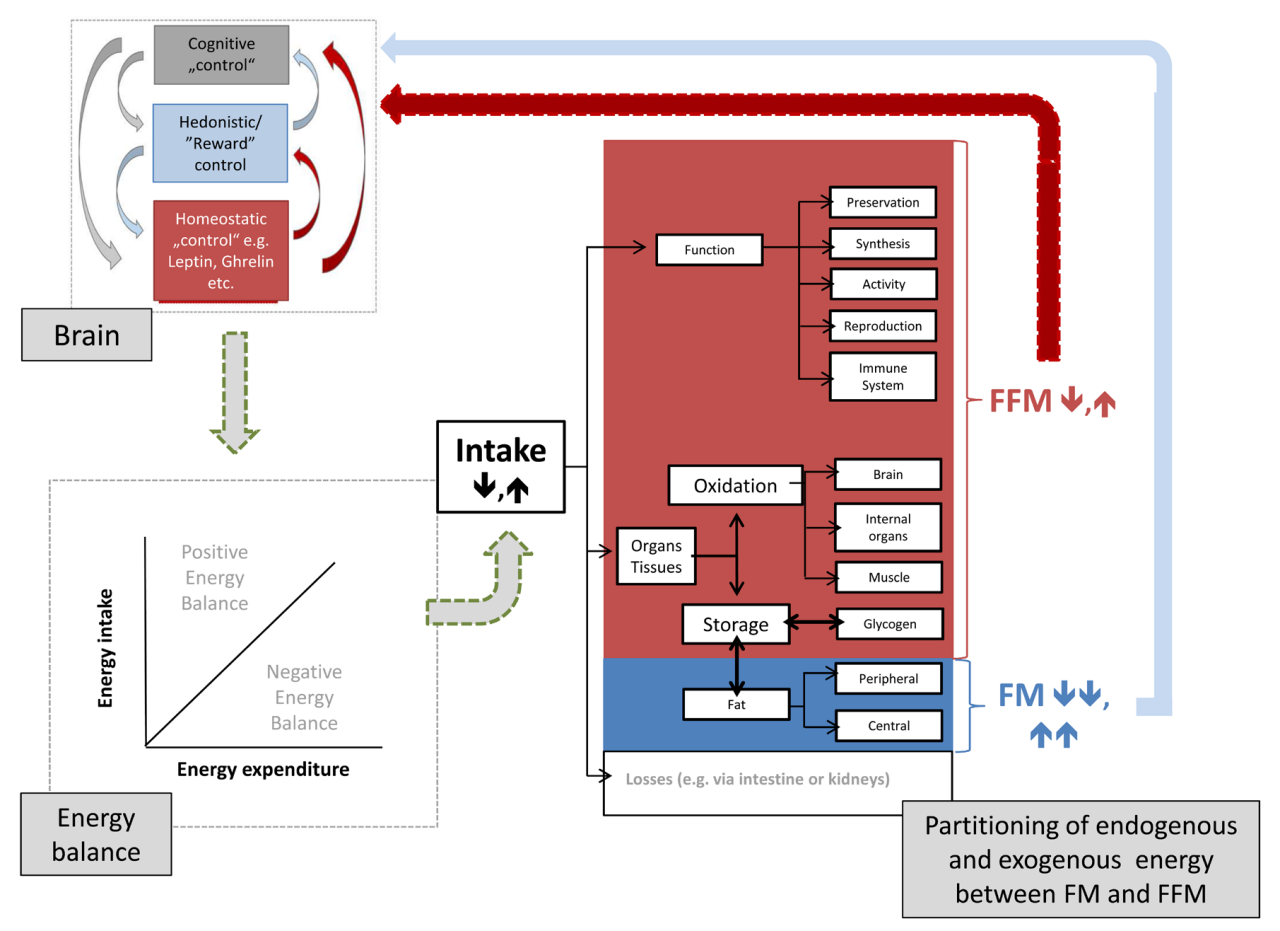
Brain control of energy balance and effects of low or high energy intake on partitioning of endogenous and exogenous energy to and from fat mass (FM) and fat free mass (FFM). FFM and FM both exert feedback controls on different levels of the brain control systems of energy balance. It is assumed that FFM is the major determinant of energy balance. See text for further details. The right part of the figure (i.e. the partitioning model) is based on the original work of Jonathan Wells (see 83).

As far as metabolic adaptation during early weight loss is concerned, another threshold (or a low set point) related to the depletion of hepatic glycogen stores has been proposed ^69^. This finding is in line with the idea that energy allocation to the brain controls EE (i.e. the brain is assumed to have a hierarchical position in whole-body energy metabolism ^75^). The brain has a high metabolic rate ^14, 76^ and is the only organ which does not lose weight with weight loss ^13^. When compared with muscle-specific metabolic rates, the brain and skeletal muscle differ by a factor of 18 ^76^. Since the brain demands a constant energy budget, it has “pole position” in a competitive situation of whole-body energy allocation and thus control of whole-body EE ( Figure 2).

Sedentary behavior and physical activity level also impact the control of appetite, satiety, and body weight. Becoming sedentary does not downregulate EI, i.e. at low physical activity there is a weak coupling between EE and EI ^16, 77, 78^. By contrast, an increase in physical activity improves satiety signaling ^16, 77^ and also increases activity EE (AEE). Then both REE and AEE drive EI ^77^. In fact, following the spectrum from low to high physical activity, there are “regulated” and “non-regulated” areas of EI ^77^. The association between physical activity and EI is J-shaped ^16^. However, in clinical practice, physical activity (or even exercise) does not add much to the treatment of obese patients ^79, 80^. This paradox is explained by compensations in the individual components of EE. EE increases with low and moderate physical activity but plateaus at high activities to maintain EE within the target range (that is, EE is constrained with respect to physical activity ^81, 82^). In fact, with a high level of exercise, the compensation is explained by a reduced REE, which resembles a decrease in basal biological functions, e.g. reproduction.

### A concept that needs reconsideration: is there a control of body weight or a control of energy expenditure?

The finding of a tight control of EE gives rise to speculation that body weight control (if it exists) is about control of EE ^14^. This idea provides an alternative paradigm, which is a putative set point or dual intervention point model of EE. The latter framework is characterized by a lower boundary (or lower set point of EE) given by the lowest metabolic rate needed for survival, whereas the upper set point of EE is explained by maximum mitochondrial capacity of cells of the body. If this EE–set point paradigm holds true, this would put recent concepts of body weight homeostasis into perspective. Then, any change in body weight follows the variance of EI at a tightly controlled EE, i.e. body weight itself is not controlled but results from the balance between a tight control of EE at loosely controlled EI.

### Need to go beyond the adipocentric view

During the last 25 years, most research on body weight control has been adipocentric. However, since FM accounts for only 10 to 40% of body weight, any control of FM can represent only a similarly sized part of the body weight control. In addition, FM (as far as it can be assessed today) is constant throughout a day, and it starts to decline within 72 hours in response to starvation ^13^. Thus, FM by itself (as far as it’s mass can be measured by todays methods) has no association with daily meal frequency and short-term calorie restriction ^1^. However, since decreases in plasma leptin concentrations observed with caloric restriction precede and exceed the changes in FM ^13^, the adipocentric view of body weight control refers to both the size and the secretory activity of FM.

It is only recently that, besides FM, FFM came into the center of research on body weight control ^16, 77, 78, 84, 85^. Obviously, there is need for a broader view on the control of EB and body weight taking into account biological inputs from sensory systems (i.e. taste and olfactory signals), the gut and feedback related to FFM (controlling protein and glycogen content) and FM (controlling fatness), and the role of hedonism and rewards in the contexts of environmental and behavioral pressures ^76^. Comparing different inputs, tonic afferences from FFM (signaling energy demands and metabolic requirements) and FM (signaling ES) have to be differentiated from episodic or dynamic feedback from the gut (signaling nutrient availability and meal and macronutrient intake by neural and enteroendocrine signals) ^16^.

### Need to address homeostatic as well as hedonic aspects ( Figure 2)

EI is regulated by interactions between homeostatic and non-homeostatic mechanisms; it is thus influenced by experiences, learning, and culture ^86^. This helps to explain why individual factors (like leptin and insulin) may have limited effects on EI. Using functional MRI (fMRI) after intra-nasal insulin application, selective insulin resistance in the prefrontal cortex (responsible for cognitive control and decision making) and in the hypothalamus was characterized as being associated with reduced inhibition of EI, food craving, and thus overeating in obese patients ^87^. In addition to the “cognitive brain”, hedonic and incentive signals related to brain reward systems of the “emotional brain” (related to the mesoaccumbal dopamine system) may further add to overeating (i.e. eating is pleasurable and rewarding). In line with this idea, two set points, a homoeostatic and a hedonic set point, have been proposed, with obesity affecting the balance between the two and one inducing shift in the other ^88^. Going on with that idea, “metabolic obesity” (with a genetically determined set point) and “hedonic obesity” (due to hedonic overeating overriding the homoeostatic set point) has been defined, and the two types of obesity may serve as a future stratification in the treatment of obese patients ^89^. However, the quantitative effects of non-homeostatic influences on the set point model are unknown.

## A self-critical view at the end

The ideas presented in this paper also point to the need for a self-critical view: two generations of scientists might have gone the wrong way when they (i) followed a hypothetical concept (i.e. there is biological control of body weight), (ii) had to accept the limited promise of methodologies to assess EB, and (iii) focused too much on statistical associations (e.g. calculating heritability estimates of BMI and, in the case of GWAS, studying associations between allele frequencies and crude anthropometric phenotypes) without addressing detailed and sound concepts and targeted analyses of structures and different levels of body weight control. It is also worthwhile to keep in mind that our present thinking is based not only on objective data but also on the interpretation of scientists, which adds a subjective factor to the discussion related to intelligence and the sovereignty of interpretation ^55^.

Faced with the present lack of direct evidence for the biological control of body weight in humans, there is need of (i) conceptual thinking, (ii) better methods to be developed in integrative physiology, and (iii) controlled (instead of merely observational) studies. It is a principal matter of science that we should also be open to the alternative idea, i.e. there is no feedback control of body weight and thus a set point does not exist with multiple settling points to explain weight changes. Since no model can perfectly explain weight changes in humans, this may suggest the possibility of some misconception of past and present research activities on body weight homeostasis.

It is obvious from the present state of the art that observational and poorly controlled studies are not a sufficient basis to form reliable knowledge and guidelines regarding body weight control ^55^. Back to the starting line again, one may also take an evolutionary point of view (which is frequently taken as justification when discussing body weight homeostasis) and ask a simple question: what should be the advantage of a tight control of body weight? As far as the body weight–mortality association is concerned, the normal range of body weight is broad, i.e. a variance of 20 kg does not affect cardio-metabolic risk. Thus, except for extreme body weights (as seen in severely obese patients or *vice versa* with underweight and malnutrition), an advantage of a tight control of body weight or FM within a broad normal range is unlikely to exist. Biologically, a normal range (if it exists) is difficult to define. Accepting a lower boundary, a one-intervention point model with multiple settling points (or equilibria) above a low and critical body weight (or low body fat and/or low-protein and/or low-glycogen content in the liver associated with the risk of hypoglycemia) which is related to an increased risk of impaired body functions, infectious diseases, and ultimately death, may provide an alternative model to be discussed in future.

To summarize, presently, there are three different models of body weight control. Although striking at first view, all models have limitations and cannot fully explain weight fluctuations in humans. In the short term, there is no auto-correlation between EI and EE, which might argue against a tight control system. Long-term control of body weight may suit the settling point model. The present evidence suggests that biological control (if it exists) is more likely to become apparent in normal-weight subjects and during caloric restriction and weight loss. However, there is obvious need of (i) an open discussion between scientists about shortcomings in past and present research and (ii) some food for thought about better concepts, methods, and research on body weight homeostasis in the future.

## Abbreviations

AEE, activity energy expenditure; AT, adaptive thermogenesis; BIA, bioelectrical impedance analysis; BMI, body mass index; DIT, diet-induced thermogenesis; DXA, dual energy absorptiometry; EB, energy balance; EE, energy expenditure; EI, energy intake; ES, energy stores; FFM, fat free mass; FM, fat mass; FTO, fat mass and obesity-associated gene; GWAS, genome-wide association studies; REE, resting energy expenditure; SNS, sympathetic nervous system; VAT, visceral adipose tissue; WC, waist circumference.

## Footnotes

^1^ Heritability is a statistical concept that draws upon correlations between relatives to quantify how much of the overall variability of a phenotype at the population level is due to genetic variation. For example, a heritability of 0.5 for body weight would imply that half of the weight difference between two unrelated individuals is directly or indirectly attributable to genetic differences between them ^24^.
